# Effects of orthokeratology lenses on tear film and tarsal glands and control of unilateral myopia in children

**DOI:** 10.3389/fcell.2023.1197262

**Published:** 2023-06-21

**Authors:** Li Li, Taichen Lai, Jing Zou, Linling Guo, Zhiming Lin, Jiawen Lin, Ying Xue

**Affiliations:** ^1^ Shengli Clinical Medical College of Fujian Medical University, Fuzhou, China; ^2^ Ophthalmology Department, Fujian Provincial Hospital South Branch, Fujian Provincial Hospital, Fuzhou, China; ^3^ Division of Ophthalmology, Department of Surgery, University of Melbourne, Melbourne, VIC, Australia; ^4^ Centre for Eye Research Australia, Royal Victorian Eye and Ear Hospital, Melbourne, VIC, Australia; ^5^ Department of Clinical Medicine, Fujian Medical University, Fuzhou, China; ^6^ College of Computer and Big Data, Fuzhou University, Fuzhou, China

**Keywords:** myopia, orthokeratology lens, tear film, tarsal gland, artificial intelligence

## Abstract

**Introduction:** To investigate the effects of an orthokeratology lens on the tear film and tarsal glands and myopia control in children with unilateral myopia using an intelligent analysis model.

**Methods:** We retrospectively reviewed the medical records from November 2020 to November 2022 of 68 pediatric patients with unilateral myopia in Fujian Provincial Hospital who had been wearing an orthokeratology lens for more than 1 year. The 68 myopic eyes were included in the treatment group, while the 68 healthy, untreated contralateral eyes were included in the control group. Tear film break-up times (TBUTs) were compared between the two groups at various intervals, and an intelligent analysis model was used to compare the deformation coefficients of 10 meibomian glands in the central area and the different positions of the glands in the two groups after 12 months of treatment. Changes in axial length and equivalent spherical power were also compared between the groups before and after 12 months of treatment.

**Results:** In the treatment group, TBUTs differed significantly between 1 and 12 months after treatment, although no significant differences from baseline were observed at 3 or 6 months. No significant differences in TBUTs were observed at any time point in the control group. After 12 months of treatment, significant between-group differences were observed for glands 2, 3, 4, 5, 6, 7, 8, and 10 (numbered from the temporal to nasal regions). The treatment group also exhibited significant differences in deformation coefficients at different detection positions in the central region, with glands 5 and 6 exhibiting the highest deformation coefficients. Increases in axial length and equivalent spherical power were significantly greater in the control group than in the treatment group after 12 months of treatment.

**Discussion:** Wearing orthokeratology lenses at night can effectively control myopia progression in children with unilateral myopia. However, long-term use of these lenses may lead to meibomian gland deformation and impact tear film function, and the extent of deformation may vary at different positions in the central region.

## 1 Introduction

In recent decades, technological advancements have introduced a variety of electronic products and children’s toys that are viewed within a close range and have become ubiquitous in daily life. The childhood developmental period is a critical window for both cognitive and physical growth. Increased emphasis on educational attainment has resulted in a high frequency of close-range eye behaviors (e.g., reading, mobile device usage, and computer usage). When coupled with improper reading and writing postures, this can lead both eyes to develop different degrees of myopia during eye development. Accordingly, one eye may develop myopia earlier than the other, resulting in unilateral myopia. Children with unilateral myopia present with overload of the myopic eye when viewing near objects, which can aggravate the progression of myopia and lead to symptoms of ocular fatigue due to decreased coordination between the eyes. If the difference in the refractive error between the two eyes is greater than 2.5 D, fine motor impairments can be observed, and children can find it difficult to adapt to wearing frame glasses, thereby affecting quality of life.

The orthokeratology lens, which is a reverse geometry lens made of highly gas permeable rigid material, is worn by patients on the corneal surface during sleep. The central region of the cornea is flattened by the positive pressure of the base curve at the central region of the lens, allowing epithelial cells to accumulate at the reverse curve through migration, thereby effectively halting the development of myopia. At present, the orthokeratology lens is an effective method for controlling disease progression in children with low to moderate myopia, and the strategy has recently gained attention given the relative comfort of the lens and low risk of complications (Müller et al., 2016). In addition to delaying myopia progression during adolescence, several studies have demonstrated (Ren et al., 2016; Xie and Guo, 2016) that orthokeratology lenses help to control the rapid growth of the eye axis and negate the need for frame glasses. However, long-term use of an orthokeratology lens may exert detrimental effects on the tear film and tarsal glands via mechanical irritation or hypoxic interference.

To address this gap in knowledge and determine the effectiveness of myopia control, the present retrospective study was designed to investigate the effects of orthokeratology lenses on the tear film and tarsal glands in children with unilateral myopia. To achieve this aim, we analyzed complete data of pediatric patients treated at our hospital who had been wearing an orthokeratology lens in one eye continuously for more than 12 months using an intelligent analysis model.

## 2 Materials and methods

### 2.1 General information

We reviewed the medical records from November 2020 to November 2022 of 68 pediatric patients with unilateral myopia treated in the Department of Ophthalmology of Fujian Provincial Hospital. The myopic eyes of patients treated using an orthokeratology lens were included in the treatment group (68 eyes), in which the mean spherical equivalent was −1.92 ± 1.21 D, whereas the healthy, untreated contralateral eyes were included in the control group (68 eyes), in which the mean spherical equivalent was +1.06 ± 0.68 D. The inclusion criteria were as follows: a) age of 8–14 years, b) more than 1 year of complete data for review, c) diagnosis of unilateral myopia with a spherical equivalent of −1.00 to 6.00 D in the myopic eye, d) best corrected visual acuity ≥5.0 in both eyes, and e) normal intraocular pressure and fundus in both eyes. The exclusion criteria were as follows: a) organic eye disease; b) corneal diseases, moderate or severe allergic conjunctivitis, dry eye disease, or keratoconus; c) poor compliance and/or inability to be followed up according to medical advice; d) combined use of other myopia interventions, such as low-dose atropine and defocus lenses; and e) contraindications to orthokeratology lens use. The risks and potential complications of wearing the orthokeratology lens were explained in detail to the patients and guardians before treatment, and consent was obtained for fitting. The study was approved by the Ethics Committee (K2020-03-124) of the hospital, and the parents or guardians of the enrolled patients signed an informed consent form.

### 2.2 Methods

#### 2.2.1 Fitting for the orthokeratology lens

All patients underwent appropriate eye examinations prior to enrolment and fitting, including naked eye vision examination, best corrected visual acuity, mydriatic retinophotoscopy, specular microscopy, corneal topography, IOLMaster examination, slit lamp examination, tear film break-up time (TBUT) estimation, and infrared photography of the tarsal glands. Patients eligible for the orthokeratology lens were screened with reference to the inclusion criteria. A suitable trial orthokeratology lens was selected for try-on and evaluation based on the corneal diameter and corneal topography of each patient. After 30 min of trial fitting, the dynamic and static fit of the lens was evaluated, and the parameters of the trial lens were adjusted until the ideal fit was achieved. A lens with ideal fit was defined as a well-centered lens with 1–2 mm of mobility when blinking, with all curves stained to standard. All patients were instructed by the same optometrist on the standard methods of lens removal, placement, and care. The minimum and maximum durations of nighttime lens wear were set to 8 h and 10 h, respectively.

#### 2.2.2 Method of constructing an intelligent model for the tarsal glands

**FIGURE 1:**
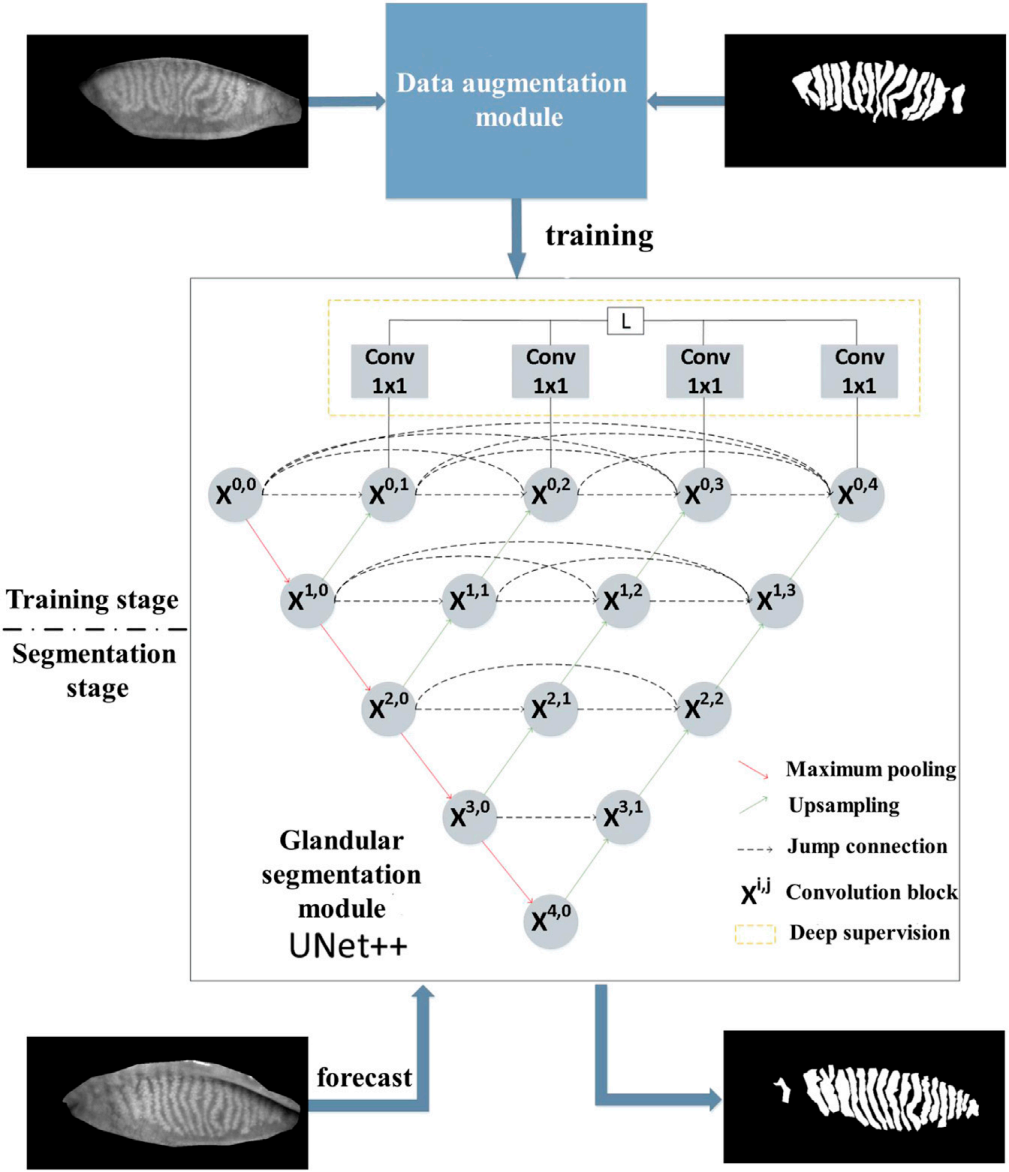
The procedure for meibomian gland segmentation based on UNet++.

UNet++ was introduced to construct a tarsal gland segmentation model and provide a workflow for automatic gland segmentation, as shown in Figure 1. We considered tarsal gland segmentation as a binary classification problem at each pixel of an image. Tarsal gland segmentation was divided into two stages (training and segmentation) and included the following three modules.

##### 2.2.2.1 Data augmentation module

In this module, an automatic data augmentation strategy was introduced to randomly select *N* of 11 types of data augmentation methods (e.g., cropping, flipping, cutting, translation, rotation, equalization, contrast adjustment, brightness adjustment), following which the corresponding magnitude of transformation, *M*, for these *N* types was selected. Eventually, we set *N* to 2 and the selection range for *M* to 1–10 based on experimental findings. The data augmentation module used for this study did not require a manually designed data augmentation strategy, could provide adequate data samples for the gland segmentation model, and enhanced the generalizability of the model.

##### 2.2.2.2 Gland segmentation module

The infrared images of the tarsal glands were grayscale images, which do not contain rich semantic information. Our preliminary analyses indicated that it would be inappropriate to use a complex network model. Proposed in 2015, the UNet model (Ronneberger et al., 2015) was designed specifically for application in medical image segmentation. UNet uses hop connections to merge the superficial and deep semantic feature maps to overcome the information loss caused by subsampling, thereby significantly improving the accuracy of medical image segmentation. UNet++ is an improved version of UNet in which the hop connections have been redesigned to further reduce the semantic gap in merging the features between encoder and decoder. This module was used to introduce the UNet++ model as the main network for automatic gland segmentation.

##### 2.2.2.3 Gland analysis module

After segmenting the tarsal glands, a small portion of the automatically segmented images can be processed again to capture missed areas or select additional areas using the editing tool in the software. After perfecting the tarsal gland images, the parameters of the gland model were analyzed, and the deformation coefficients of the glands (reflecting the degree of gland deformation) were automatically calculated. The deformation coefficient was calculated as follows:

$$\frac{p_{a}\times p_{b}}{length{\left(central\right)}^{2}}\times \left(\frac{\sqrt{\sum_{i=1}^{n}{\left(w_{i}-w_{avg}\right)}^{2}}}{n}+1\right)$$

**FIGURE 2:**
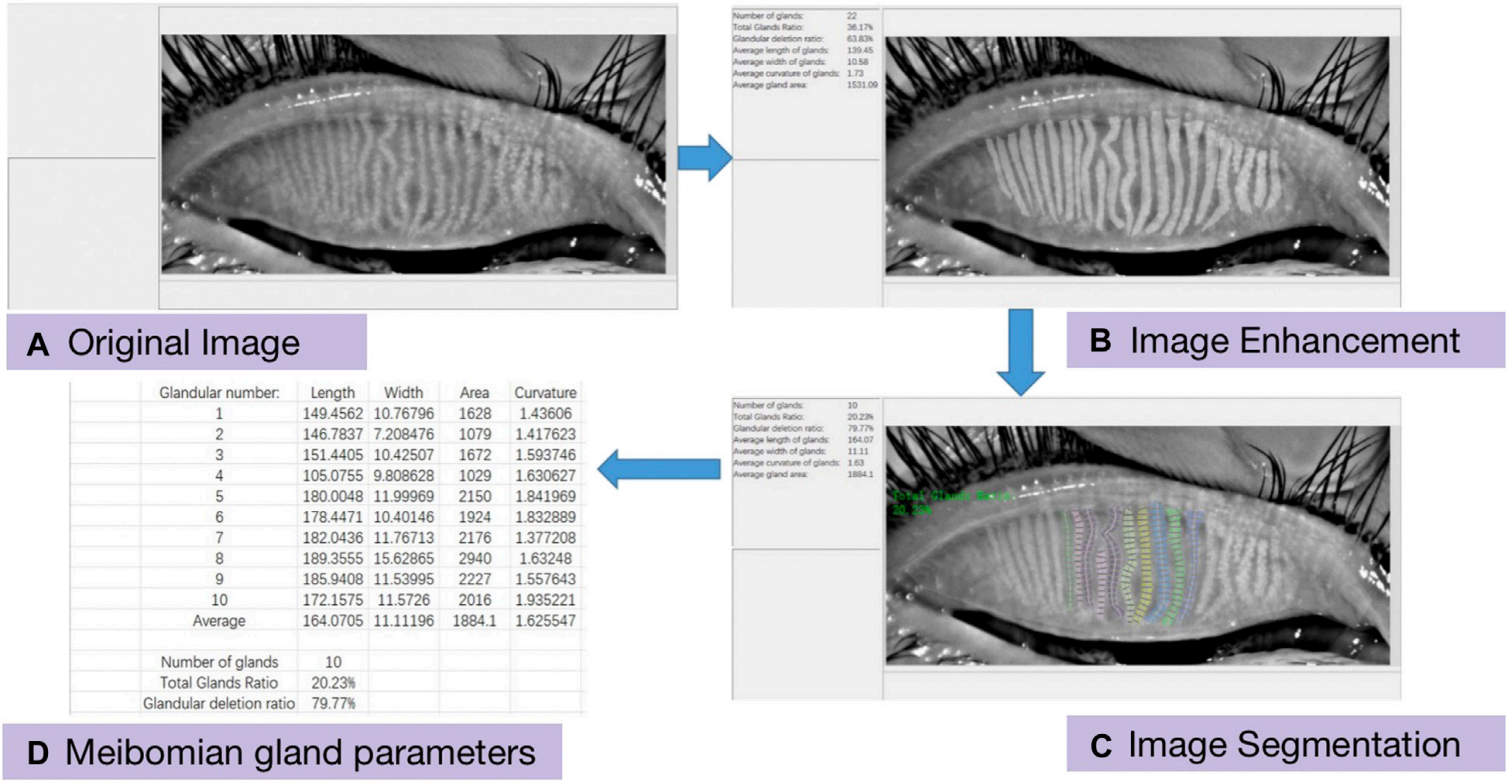
Prediction images to be edited.

The formula for calculating the deformation coefficient of the gland was established and improved upon based on the arc-string ratio model (Figure 2). Here, $p_{a}$ refers to the length of the left side of the gland, $p_{b}$ refers to the length of the right side of the gland, $w_{i}$ refers to the diameter of the gland taken after each step, $w_{avg}$ refers to the average diameter of the gland, length (central) refers to the length of the central line, *n* refers to the number of diameters taken by the gland, the minimum value of the formula is 1, and the deformation coefficients are dimensionless units.

### 2.3 Observational indicators

All enrolled patients underwent an eye examination before treatment (when the custom-made orthokeratology lens arrived), which was considered the starting point of the observation period. Follow-ups were conducted 1, 3, 6, and 12 months after initiating treatment. Indicators evaluated in this study included TBUT, axial length, and spherical equivalent, as well as adverse reactions during the treatment period.

#### 2.3.1 TBUT

The inferior conjunctival sacs of both eyes were lightly touched with a fluorescein sodium strip that had been moistened with saline. The patient was asked to blink three times to allow the cornea to make full contact with the fluorescein, and the corneal condition was observed under cobalt blue light with a slit lamp. During the examination, the patient was advised not to blink until a black spot appeared on the cornea. The time taken for the first black spot to appear during the examination was recorded, and the measurement was repeated three times to obtain an average value.

#### 2.3.2 Tarsal gland deformation coefficient

Changes in the morphology of each tarsal gland were analyzed using the intelligent model. In this study, the deformation coefficients of 10 glands in the central regions of the upper eyelids of both eyes were calculated after 12 months of treatment. Using the midline of the overall tarsal gland area of each patient’s upper eyelid as the center, values were calculated for five glands extending towards the nasal and temporal sides, respectively, resulting in a total of 10 glands for analysis. The 10 glands were numbered such that the first tarsal gland at the temporal side was considered gland 1, while the first tarsal gland on the nasal side was considered gland 10.

#### 2.3.3 Axial length

IOLMaster was used to examine axial length, and the measurement was repeated five times to obtain an average value.

#### 2.3.4 Spherical equivalent

The patients were administered 1% compound tropicamide eye drops to fully paralyze the ciliary muscle. Treated eyes underwent optometry using an automatic computerized refractometer while the orthokeratology lens was in place, while control eyes underwent the same assessment on the naked eye. The measurement was repeated five times to obtain an average spherical equivalent value.

### 2.4 Statistical analysis

Data were statistically analyzed using SPSS 22.0 software. Independent samples t-tests were used to compare the average values of the two groups at each time point. Paired t-tests were used to compare the average values of the same group at different time points. Paired samples t-tests were also used to evaluate the deformation coefficients of the 10 tarsal glands in the central region in the two groups after 12 months of treatment. A one-way analysis of variance (ANOVA) was used to evaluate the deformation coefficients of the tarsal glands at different examination locations in each of the two groups. The measurement data of both groups were expressed as mean ± standard deviation (x ± s), whereas the count data were expressed as proportions (%). A difference was considered statistically significant when *p* < 0.05.

## 3 Results

Data were retrospectively analyzed for 68 patients (30 boys, 38 girls; mean age: 11.12 ± 0.76 years; age range: 8–14 years). The characteristics of the included patients are summarized in Table 1.

**TABLE 1:** Patient information.

Patient information		Number of cases
Sex	Female	38
	Male	30
Residence	Urban	52
	Rural	16
Myopic eye	Right	29
	Left	39
Premature infant	Yes	10
	No	58
Family history of unilateral myopia	Yes	4
	No	64

### 3.1 TBUT

In the treatment group, there were no statistically significant differences in TBUT from baseline at 3 or 6 months after treatment (*p* > 0.05). However, TBUTs differed significantly between 1 and 12 months after treatment (*p* < 0.05). No significant differences in TBUT from baseline were observed at any time point in the control group (*p* > 0.05) (Table 2).

**TABLE 2:** Comparison of tear film break-up time before and at 1, 3, 6, and 12 months after treatment initiation in the treatment and control groups.

Time point	Group	TBUT		t	p
		Pre-treatment	Post-treatment		
1 month	Treatment group	**10.50 ± 1.40**	**6.50 ± 0.97**	**45.445**	**.000**
	Control group	10.98 ± 1.62	10.12 ± 1.5	0.764	.272
3 months	Treatment group	10.50 ± 1.40	10.00 ± 0.82	1.785	.079
	Control group	10.98 ± 1.62	10.58 ± 1.81	.268	.725
6 months	Treatment group	10.50 ± 1.40	9.67 ± 1.5	1.132	.128
	Control group	10.98 ± 1.62	10.77 ± 1.87	.000	1.000
12 months	Treatment group	**10.50 ± 1.40**	**7.17 ± 1.36**	**29.807**	**.000**
	Control group	10.98 ± 1.62	10.85 ± 1.35	.000	1.000

Data are expressed as mean ± standard deviation.

TBUT, tear film break-up time.

### 3.2 Differences in Deformation Coefficients of 10 glands in the central regions of the upper eyelids between the Two Groups

Significant between-group differences in deformation coefficients were observed for glands 2, 3, 4, 5, 6, 7, 8, and 10 in the central region (*p* < 0.05). In all cases, deformation coefficients were higher in the treatment group than in the control group (Table 3).

**TABLE 3:** Differences in gland deformation coefficients between the groups.

		Paired difference					t	Significance (two-tailed)
		Average value	Standard deviation	Standard error of the mean	95% confidence interval for difference			
					Upper limit	Lower limit		
Pair 1	Deformation coefficient of gland 1 in the treatment group—deformation coefficient of gland 1 in the control group	0.121	0.384	0.064	−0.009	0.251	1.888	0.067
Pair 2	Deformation coefficient of gland 2 in the treatment group—deformation coefficient of gland 2 in the control group	0.420	1.016	0.169	0.076	0.764	2.481	0.018
Pair 3	Deformation coefficient of gland 3 in the treatment group—deformation coefficient of gland 3 in the control group	1.207	1.544	0.257	0.685	1.730	4.691	0.000
Pair 4	Deformation coefficient of gland 4 in the treatment group—deformation coefficient of gland 4 in the control group	2.195	2.919	0.487	1.208	3.183	4.512	0.000
Pair 5	Deformation coefficient of gland 5 in the treatment group—deformation coefficient of gland 5 in the control group	4.563	3.536	0.589	3.367	5.759	7.744	0.000
Pair 6	Deformation coefficient of gland 6 in the treatment group—deformation coefficient of gland 6 in the control group	4.960	5.345	0.891	3.152	6.769	5.569	0.000
Pair 7	Deformation coefficient of gland 7 in the treatment group—deformation coefficient of gland 7 in the control group	2.205	2.062	0.344	1.508	2.903	6.417	0.000
Pair 8	Deformation coefficient of gland 8 in the treatment group—deformation coefficient of gland 8 in the control group	0.634	1.165	0.194	0.240	1.028	3.266	0.002
Pair 9	Deformation coefficient of gland 9 in the treatment group—deformation coefficient of gland 9 in the control group	0.077	0.439	0.073	−0.072	0.225	1.049	0.301
Pair 10	Deformation coefficient of gland 10 in the treatment group—deformation coefficient of gland 10 in the control group	0.268	0.343	0.057	0.151	0.384	4.677	0.000

### 3.3 Differences in Deformation Coefficients of 10 glands in the central regions of the upper eyelids at Different Examination Sites

#### 3.3.1 Treatment group

**FIGURE 3:**
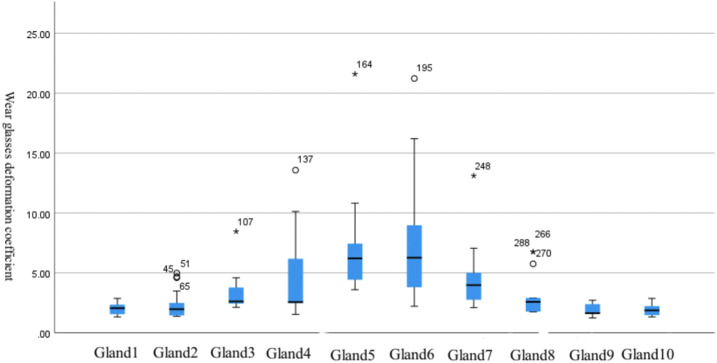
Differences in gland deformation coefficients at different detection locations in the treatment group.

Within the treatment group, significant differences in gland deformation coefficients were observed across different examination sites. Deformation coefficients for glands 5 and 6 were higher than those for glands 3 and 4, while deformation coefficients for glands 3 and 4 were higher than those for glands 1, 2, 8, 9, and 10 (Table 4; Figure 3).

**TABLE 4:** Differences in gland deformation coefficients at different detection sites in the treatment group.

Examination site	Average value	Standard deviation	95% confidence interval for the average value		F	Significance	Least significant difference (LSD)
			Upper limit	Lower limit			
Gland 1	1.983	0.454	1.830	2.137	29.003	0.000	5, 6 > 3, 4 > 1, 2, 8, 9, 10
Gland 2	2.179	0.963	1.853	2.505			
Gland 3	3.285	1.270	2.855	3.714			
Gland 4	4.120	2.894	3.141	5.099			
Gland 5	6.608	3.424	5.449	7.767			
Gland 6	7.447	4.500	5.925	8.970			
Gland 7	4.176	2.136	3.453	4.899			
Gland 8	2.684	1.248	2.261	3.106			
Gland 9	1.852	0.469	1.693	2.011			
Gland 10	1.890	0.457	1.736	2.045			

#### 3.3.2 Control group

**FIGURE 4:**
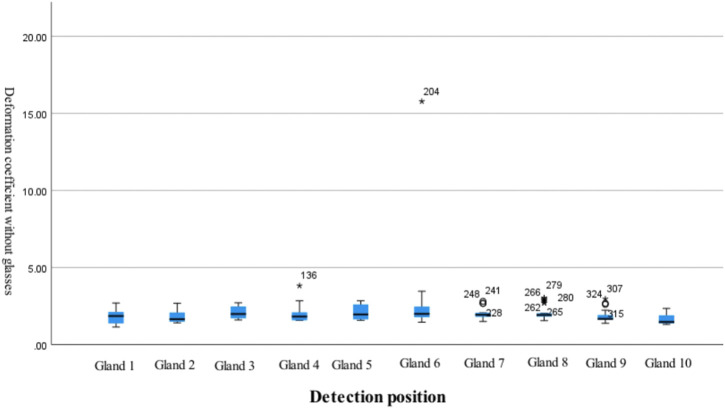
Differences in gland deformation coefficients at different detection locations in the control group.

Within the control group, significant differences in gland deformation coefficients were also observed at different examination sites (*p* < 0.05). The deformation coefficient of gland 6 was higher than the deformation coefficients for glands 1, 2, 3, 4, 5, 7, 8, 9, and 10, while the deformation coefficients for glands 3, 5, and 8 were higher than the deformation coefficient for gland 10 (Table 5; Figure 4).

**TABLE 5:** Differences in gland deformation coefficients at different detection sites in the control group.

Examination site	Average value	Standard deviation	95% confidence interval for the average value		F	Significance	Least significant difference (LSD)
			Upper limit	Lower limit			
Gland 1	1.862	0.404	1.726	1.999	3.011	0.002	6 > 1.2,3,4,5,7,8,9,10;3, 5, 8 > 10
Gland 2	1.759	0.335	1.646	1.873			
Gland 3	2.077	0.426	1.933	2.222			
Gland 4	1.925	0.447	1.773	2.076			
Gland 5	2.045	0.411	1.906	2.184			
Gland 6	2.487	2.328	1.699	3.274			
Gland 7	1.971	0.275	1.878	2.063			
Gland 8	2.050	0.437	1.902	2.197			
Gland 9	1.775	0.359	1.653	1.897			
Gland 10	1.622	0.284	1.527	1.718			

### 3.4 Axial length

In both groups, axial length increased with time after treatment. However, after 12 months of treatment, the degree of increase in axial length was significantly lower in the treatment group (0.15 ± 0.13 mm) than in the control group (0.39 ± 0.23 mm) (*p* < 0.05) (Table 6).

**TABLE 6:** Axial length before and after treatment in the two groups at 12 months.

		Change within 12 months of treatment
Increase in axial length	Treatment group	0.14 ± 0.08
	Control group	0.42 ± 0.17
	t	−11.393
	p	<0.01

Data are expressed as mean ± standard deviation.

### 3.5 Spherical equivalent

After 12 months of treatment, the spherical equivalent increased in 20 eyes (29.4%) in the treatment group, whereas it increased in 58 eyes (85.3%) in the control group, and the difference in the spherical equivalent between the two groups was statistically significant (*p* < 0.05) (Table 7).

**TABLE 7:** Spherical equivalent before and after treatment in the two groups at 12 months.

		Change within 12 months of treatment
Change in spherical equivalent	Treatment group	0.04 ± 0.16
	Control group	0.56 ± 0.36
	t	−10.168
	p	<0.01

Data are expressed as mean ± standard deviation.

## 4 Discussion

The prevalence of myopia among school-age children in East Asia is extremely high compared with other regions. The prevalence of myopia among school-age children in East Asia is extremely high compared with other regions (Grzybowski A et al., 2020). In clinical practice, most affected children present with bilateral myopia. However, poor eye habits and other environmental factors (e.g., masking the eyes on one side when writing and tilting the body when reading/writing) can lead to inconsistencies in the distance between each eye and the viewing material. Such factors may also contribute to differences in visual acuity between the eyes (Shi et al., 2021) and an increase in the number of adolescents with unilateral myopia presenting with anisometropia. For children who have unilateral myopia with anisometropia, differences in refractive error can lead to contradictory eye adjustments and an imbalance in image size. At present, the two most common correction materials are frame glasses and orthokeratology lenses. While frame glasses are widely accepted by patients and parents in clinical practice given their convenience and low cost, correction with frame glasses is associated with problems such as imbalanced visual quality and difficulty in fusion, which will continue to increase the refractive error of myopia. In contrast, the orthokeratology lens exerts its effects via nighttime wear. In addition to controlling the rate of increase in the refractive error of myopia, orthokeratology lenses can reduce the overall refractive error of myopic eyes and improve both visual quality and fusion function in the two eyes during adolescence. Therefore, orthokeratology lens treatment has become the preferred correction method for adolescents who have unilateral myopia with anisometropia. Some studies have also shown that orthokeratology lenses are the best means to control myopia among the available non-surgical treatment methods (Walline et al., 2020).

Despite these results, Yang et al. (2021) find that Short-term OOK may reduce the stability of the tear film and increase damage to the corneal epithelium. Long-term OOK could induce ocular inflammation through the disruption of meibomian glands. However, Na et al. (2016) reported no significant changes in tear film stability after treatment with an orthokeratology lens. While TBUT was the main observational indicator in their study, the results were largely influenced by the subjective nature of the examinations and patient cooperation. To ensure more objective analyses, each patient/parent was informed of the examination precautions and the standard of cooperation required during examination. In the current study, the treatment group exhibited a significant reduction in TBUT from baseline at 1 month after treatment. This may be because the initial shaping begins in the early stage of lens wear, in which the corneal epithelial cells begin to migrate and distribute. This in turn leads to an uneven corneal surface, which affects the uniform distribution of tear film and reduces tear film stability. In contrast, TBUTs at 3 and 6 months after treatment initiation did not significantly differ from baseline. This may have been due to improvements in tear film stability following stabilization of the corneal shape, resulting in restoration of tear film function during treatment. However, some studies have found that long-term use of an orthokeratology lens may lead to altered tarsal gland function (Walline et al., 2020). Others have similarly concluded (Zhu and Huang, 2016; Zhou et al., 2019) that wearing an orthokeratology lens for 1 year exerts certain effects on the tarsal glands and that the frequency with which tear film function is examined should be increased. Gad et al. (2019) also noted that changes in the number and function of goblet cells and changes in the inflammatory response of the ocular surface may contribute to decreased tear film stability during long-term treatment with an orthokeratology lens. These findings are in accordance with the significant change in the TBUT after 12 months of treatment in our patients.

In clinical practice, although children undergoing long-term treatment with an orthokeratology lens often present no significant atrophy of the tarsal glands, other morphological changes such as distortion may emerge. Lin et al. (2020) recently reported significant correlations of tarsal gland tortuosity with the meiboscore and meibum expressibility score, suggesting that changes in the morphology of the tarsal glands can affect their function. However, at present, clinical examinations do not allow for a more accurate quantitative assessment of the tarsal glands. Therefore, we constructed an intelligent analysis model based on deep learning methods to analyze the deformation coefficients of the tarsal glands after processing and segmenting the infrared photographic report. After a preliminary test of this method, Our team, Lin et al. (2022), found that the average accuracy of the algorithm was 94.31%, with an average sensitivity of 82.15%, an average specificity of 96.13%, and an average intersection ratio of 65.55%. Based on this tarsal gland segmentation algorithm, we developed a quantitative tarsal gland analysis model that can quantitatively analyze data from each tarsal gland in the upper eyelid. However, in the current study, data were collected for only 10 tarsal glands in the central region of the upper eyelid, mainly because it is easier to collect complete data for the upper eyelid than for the lower eyelid, and the images are clearer. Further, The glands in the central region play a major role in maintaining the stability of the microenvironment at the ocular surface.

After 12 months of treatment with an orthokeratology lens, we observed significant between-group differences in the deformation coefficients of the 10 tarsal glands in the central region, with the deformation coefficients being significantly higher in the treatment group than in the control group. This result suggests that long-term use of an orthokeratology lens exerts certain effects on tarsal gland morphology. In addition, although there were certain differences between the 10 tarsal glands at different sites in both groups, the box plots indicated that the average deformation coefficients of the 10 glands in the central region were closer to each other in the control group. In the treatment group, the average deformation coefficients of the glands near the center were significantly higher than those of the glands near the periphery. This may be related to the positioning of the orthokeratology lens at the central cornea. Yu et al. (2022) reported increased atrophy of the meibomian glands in the lower eyelid in children wearing orthokeratology lenses. During sleep, a certain amount of pressure is exerted not only on the cornea but also on the tarsal glands owing to the mechanics of the orthokeratology lens, which may lead to morphological changes in the glands closer to the central area. In the future, based on the results of this study, for children with OK lenses, it is recommended that additional images of the tarsal gland be taken at the time of outpatient follow-up and that the main focus be on morphological changes of the tarsal gland in the central region.

The refractive error of the human eye is closely related to both corneal curvature and axial length. An increase in negative refractive error is mainly related to an increase in axial length when the corneal curvature remains unchanged (Alhussain et al., 2022). In this study, both the treatment and control groups exhibited an increase in axial length after 12 months. After 12 months of treatment with an orthokeratology lens, the average increase in axial length in the treatment group was 0.15 ± 0.13 mm, which was significantly less than that in the control group. The smaller change in axial length in the treatment group may be explained by a reduction in the average corneal curvature in the central region of the cornea through positive pressure exerted by the base curve of the lens. Light passing through the central region is then better concentrated at the fovea of the macula. The siphon principle of the reverse curve causes the corneal epithelial cells to migrate and accumulate at the reverse curve region, and the incident rays passing through this region are focused in front of the peripheral retina. This then forms a myopic defocus that inhibits the increase in axial length. Although the rate of increase in axial length was slower in the treatment group than in the control group of the present study, axial length was significantly longer in the treatment group than in the control group at each time point during the 12-month treatment period. This suggests that for the same patient, the axial length increases with an increase in negative refractive error, provided that the difference in corneal curvature between the two eyes is insignificant.

In clinical practice, orthokeratology lenses are usually replaced each year. If each parameter (including corneal topography positioning, corneal condition, and changes in the eye axis) remains stable at this time, many parents will choose to have their child fitted for a new orthokeratology lens. Therefore, the change in spherical equivalent after pupil dilation following use of the lens is often used to assess myopia control after 1 year of treatment. For this reason, we also chose to compare changes in the spherical equivalent after lens use between the treatment and control groups. While eyes in the control group were not initially myopic, as the treatment group wore the orthokeratology lens for a long period of time, the changes in spherical equivalent became much higher in the control group than in the treatment group. This finding is in accordance with the significantly greater increase in axial length observed in the control group.

This study has some limitations. Given the retrospective nature of the analysis, we were unable to evaluate infrared photographic data of the tarsal glands when the patients first wore the orthokeratology lens, and the abnormal morphological changes in the tarsal glands of the ipsilateral eyes before and after 1 year of wearing the lens were not further compared. These relationships should be examined in future studies.

## 5 Conclusion

In children with anisometropia, unilateral treatment with an orthokeratology lens was effective in controlling increases in axial length and negative refractive error in the myopic eye, thereby delaying the progression of myopia. Increases in axial length and negative refractive error were greater in the contralateral non-myopic eyes. The TBUT of myopic eyes tended to decrease with long-term use of the orthokeratology lens. Using an intelligent model to analyze the deformation coefficients, we found that long-term orthokeratology lens use significantly influenced morphological changes in the 10 tarsal glands in the central region, exerting a relatively greater effect on the glands closer to the central region. Given these results, children undergoing single-eye treatment with an orthokeratology lens should be closely followed up for changes in spherical equivalent, axial length, tarsal gland morphology, and tear film function in both eyes to ensure that any abnormalities are detected and addressed using appropriate interventions in a timely manner. This will not only help to control the increase in the axial length of the non-myopic eye as early as possible but also ensure better comfort and reduce the likelihood of complications with long-term use.

## Data Availability

The raw data supporting the conclusion of this article will be made available by the authors, without undue reservation.
